# Multiple introgression events from ghost Rüppell’s fox mitochondrial lineages into red fox

**DOI:** 10.1038/s41598-026-45528-8

**Published:** 2026-03-28

**Authors:** Rita Gomes Rocha, Ali Adan Hassan, Sadık Demirtaş, Mariana Meneses-Ribeiro, Joana L. Rocha, İslam Gündüz, Raquel Godinho

**Affiliations:** 1 InBIO Laboratório Associado, CIBIO–Centro de Investigação em Biodiversidade e Recursos Genéticos, Universidade do Porto, Vairão, Portugal; 2 BIOPOLIS Program in Genomics, Biodiversity and Land Planning, Centro de Investigação em Biodiversidade e Recursos Genéticos (CIBIO), Vairão, Portugal; 3 Department of Biology, Faculty of Sciences, Ondokuz Mayis University, Samsun, Türkiye; 4 Department of Molecular Biology and Genetics, Faculty of Sciences, Ondokuz Mayıs University, Samsun, Türkiye; 5 Departamento de Biologia, Faculdade de Ciências, Universidade do Porto, Porto, Portugal; 6 Department of Biology, New York University, New York, USA; 7 Department of Zoology, University of Johannesburg, Johannesburg, South Africa

**Keywords:** Anatolian Peninsula, Canidae, Ghost introgression, Mitogenomes, Phylogeny, Ecology, Ecology, Evolution, Genetics, Zoology

## Abstract

Mitochondrial introgression has been reported between red fox (*Vulpes vulpes*) and Rüppell’s fox (*Vulpes rueppellii*). While an evolutionary scenario of old divergence followed by recent mitochondrial introgression has been proposed, the directionality, prevalence, and timing of this event remain unclear. To further investigate this scenario, we analysed mitogenomes (n=85), including four newly generated red fox mitogenomes from Türkiye and a new Rüppell’s fox mitogenome from the United Arab Emirates, and partial mitochondrial DNA sequences (n=320) from both species across their ranges, including a newly comprehensive sampling in the Anatolian Peninsula (n=80). Our results are consistent with unidirectional mitochondrial introgression from the desert-adapted Rüppell’s fox into the generalist red fox, likely driven by climatic shifts promoting secondary contact and asymmetrical reproductive behaviour. Phylogenetic analysis unveiled two deeply divergent mitochondrial lineages of Rüppell’s fox sampled among red fox individuals: one distributed across Türkiye, Iran and Tunisia, and another restricted to Iran, suggesting that likely now-extinct (ghost) mitochondrial lineages may have introgressed into red foxes at least twice. Estimates of the time to the most recent common ancestor indicate that introgressed and contemporary Rüppell’s fox mitochondrial lineages diverged approximately 230 kya, predating the current intraspecific mitochondrial diversification of Rüppell’s fox lineages (~72 kya). This study highlights how comprehensive mitogenomic data and exhaustive regional surveys are critical for elucidating the complexity of introgression patterns in closely related canids.

**Supplementary Information:**

The online version contains supplementary material available at 10.1038/s41598-026-45528-8.

## Introduction

Hybridization and introgression are widespread phenomena shaping the evolutionary history of many taxa^1,2^. Mitochondrial introgression, in particular, is a recurrent phenomenon, detectable through mitonuclear discordance in genealogies and possibly led by adaptive introgression, sex-biased asymmetries and demography^3^. Specifically, range expansion is a key factor influencing the frequency of hybridization and subsequent introgression. Literature on the interplay between genetic and demographic processes has shown that when a species undergoes range expansion and comes into contact with a closely related species with which reproductive barriers are still incomplete, hybridization can lead to high levels of asymmetric introgression typically from the local species into the colonising one^4^. However, if there is sufficient intraspecific gene flow within the colonising species, genetic drift is reduced, and introgressed alleles are less likely to increase in frequency by chance^5^.

Numerous examples of introgression have been repeatedly reported within the Canidae family^6–10^, with a recent example being the mitochondrial introgression between red fox *Vulpes vulpes* (Linnaeus, 1758) and Rüppell’s fox *Vulpes rueppellii* (Schinz, 1825)^11,12^. These closely related sister species^13,14^ inhabit largely different environments, with the red fox having a broad distribution across the entire Northern Hemisphere^15^, while the Rüppell’s fox is circumscribed to the deserts of North Africa and the Middle East^16^. However, their ranges overlap in the semi-arid regions of the Sahara Desert and along the Nile River in North Africa, and across the Arabian Peninsula, extending eastwards to Pakistan^15,16^.

The first comprehensive study on the evolutionary origins and phylogeography of the red fox identified three geographically structured mitochondrial clades across the species’ range, while also revealing a fourth group of highly divergent haplotypes carried by individuals from North Africa, Middle East and East Asia, referred to as “Palearctic basal haplotypes”^17^. A more recent study targeting both red and Rüppell’s fox sister species revealed that these “Palearctic basal haplotypes” are nested within the same mitochondrial cluster of all Rüppell’s haplotypes, rendering the red fox paraphyletic^12^. To explain this mitochondrial paraphyly the authors proposed three evolutionary scenarios. Two of these hypotheses – the “Rüppell’s fox as a recently diverged red fox ecotype” and “mitochondrial incomplete lineage sorting” – were rejected by^12^ based on fossil evidence^18^, significant eco-physiological differences^19–21^ and genome-wide differentiation between the two species^14,22^ The third scenario, considered the most plausible explanation for the mitochondrial paraphyly, named “old divergence and recent introgression”, involved an ancient divergence of the two sister species at 1.15 million years ago (Mya)^17^ followed by recent secondary contact with gene flow^12^. However, the direction of the introgression remained uncertain, with both directions considered equally plausible^12^.

Phylogenetic tree topology can provide valuable insights into to the directionality of introgression, as sister relationships generate explicit expectations regarding the donor and the recipient lineages^22,23^. In this context, if introgression occurred from Rüppell’s fox into red fox, introgressed haplotypes are expected to cluster within Rüppell’s fox lineages rather than with those of the red fox. Conversely, if introgression occurred from red fox into Rüppell’s fox, the introgressed haplotypes are expected to cluster with red fox lineages instead of clustering with Rüppell’s fox.

In addition, red and Rüppell’s foxes differ fundamentally in their ecology, morphology and physiology^18,21^, which may help further clarify the mechanisms underlying this mitochondrial introgression event. Sex-biased interspecific mating could influence the directionality of crosses, specifically under different behavioural scenarios. Under the male competition hypothesis, males of the larger species are more likely to gain access to females of the smaller species^24^, whereas under the frequency-dependent assortative mating hypothesis, females settle for heterospecific males when conspecific preferred males are rare or absent^25^.

In this work, we combined newly generated and publicly available complete mitogenomes and partial mitochondrial DNA sequences from both red and Rüppell’s foxes across their ranges to investigate the directionality, prevalence, and timing of the introgression events. To estimate the prevalence of mtDNA introgression, we conducted intensive sampling in the previously overlooked Türkiye region, providing the most comprehensive sampling of red fox to date across this biogeographically significant and climatically diverse region, located at the junction of Europe, the Middle East, and Asia.

Based on the known dynamics of introgression^4^ and on the phenotypic and demographic traits of these two sister species, we hypothesize that mitochondrial introgression has been unidirectional, likely from Rüppell’s fox into red fox, with introgressed variants occurring at low frequency within red fox populations. This scenario is supported by the red fox’s greater ability for range expansion, the larger body size of its males both historically and at the present, and its generally higher population abundances^18–20^.

## Results

**Fig. 1:**
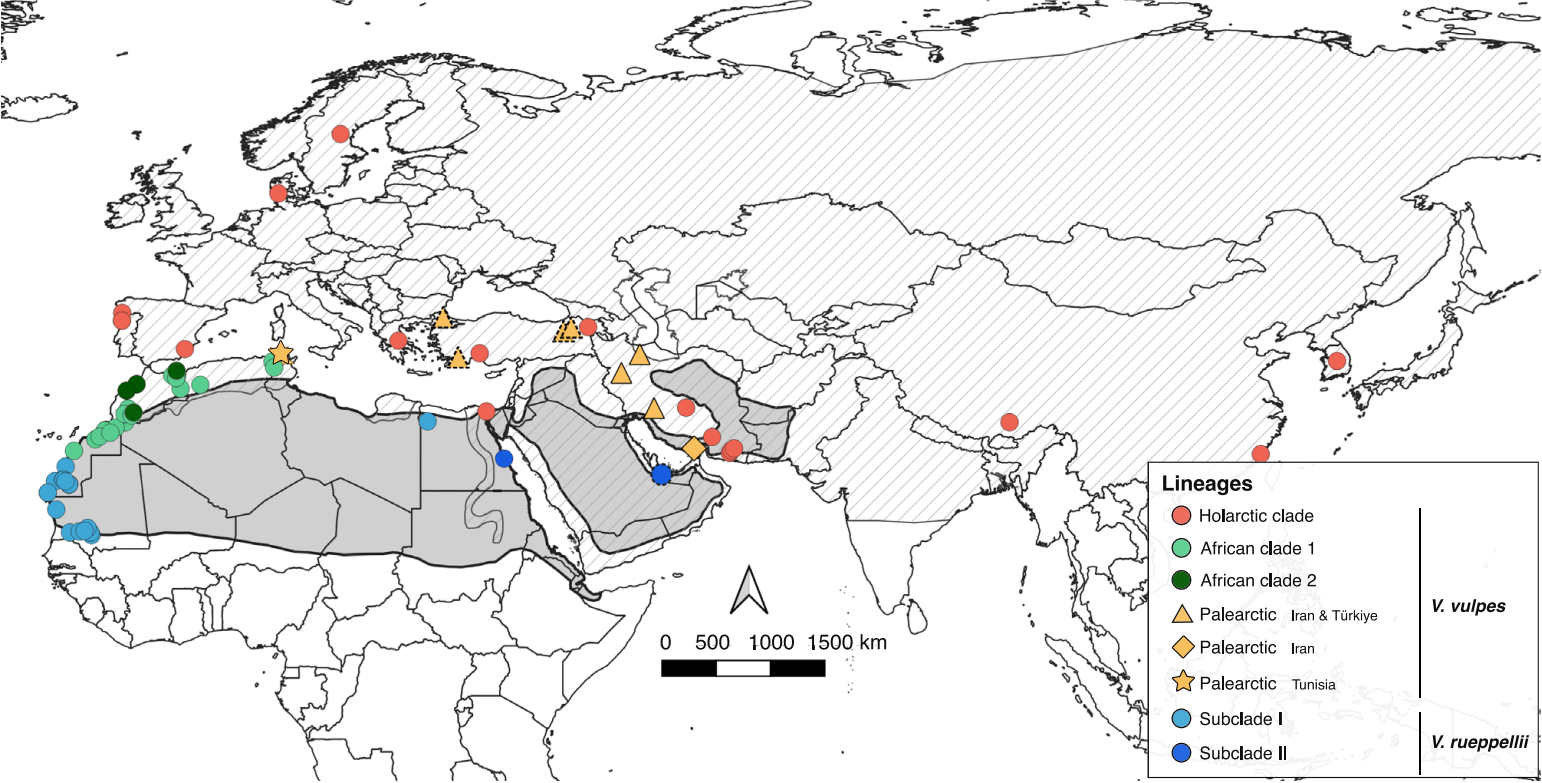
Geographic location of the 85 mitogenomes of red and Rüppell’s foxes used in this study. Colours represent maternal lineage groups obtained from the phylogenetic analysis (Figure 3). Newly generated mitogenomes are indicated by dashed outlines around the symbols. Dashed and grey areas represent the distribution of red and Rüppell’s foxes, respectively, as obtained from the IUCN^15,16^.

**Fig. 2:**
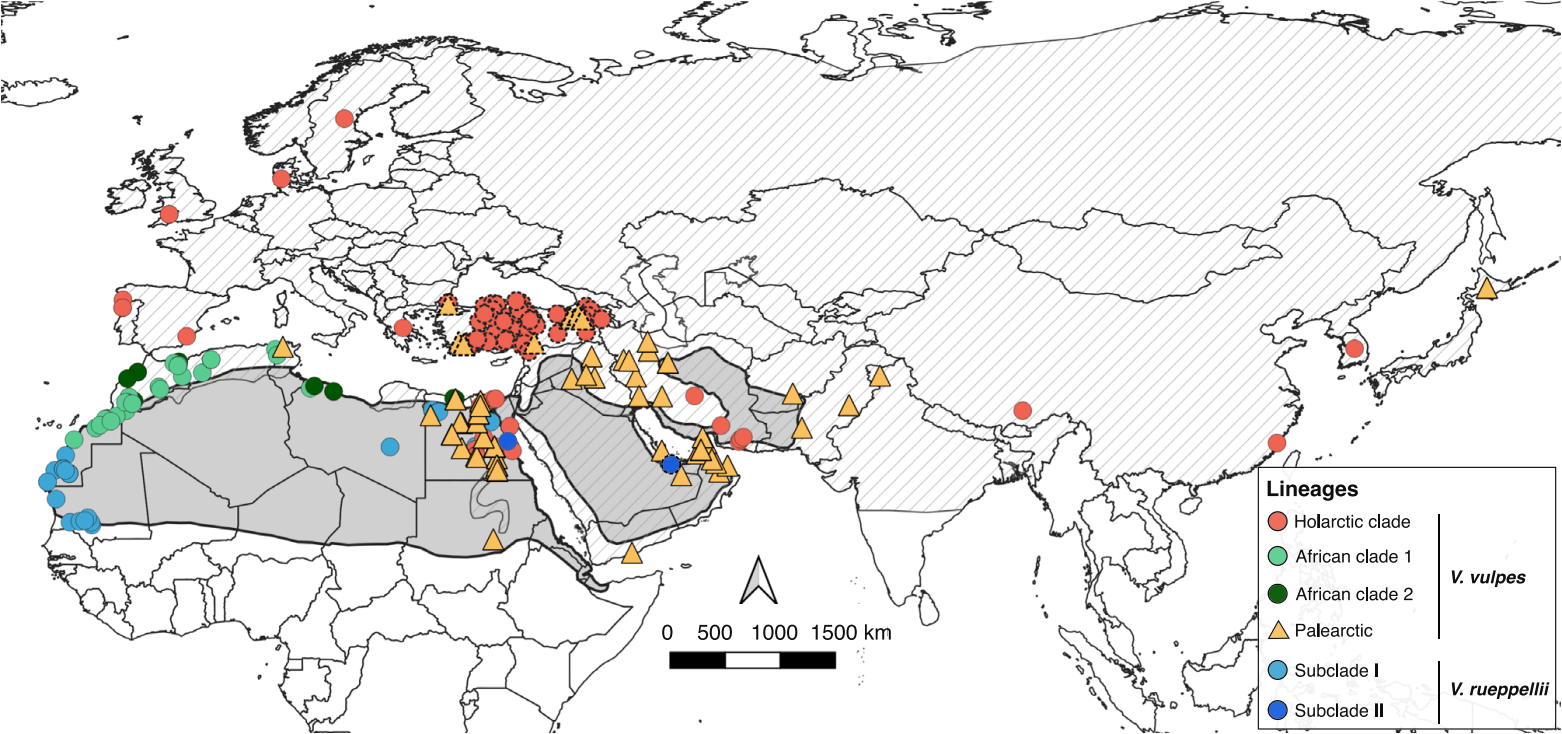
Geographic location of the 320 partial mitochondrial sequences of red and Rüppell’s foxes used in this study. Colours represent maternal lineage groups obtained from the phylogenetic analysis (Figure 3). Newly generated partial mitochondrial sequences are indicated by dashed outlines around the symbols. Dashed and grey areas represent the distribution of red and Rüppell’s foxes, respectively, as obtained from the IUCN^15,16^.

In this study, we combined four newly sequenced red fox mitogenomes from Türkiye and a new Rüppell’s fox mitogenome from the United Arab Emirates (UAE) with publicly available data to create a multiple sequence alignment comprising 16,116 bp of 85 mitogenomes of the two species (Figure 1 and Table S1). Additionally, we generated 80 partial mtDNA sequences for the red fox from Türkiye (Figure S1), representing 49 different haplotypes, which were combined with 240 additional publicly available sequences to produce a partial mtDNA alignment of 637 bp (Figure 2, Table S2). Both mitogenomes and partial mtDNA sequences showed no premature stop codons, and polymorphic sites are evenly distributed across the sequences, supporting the absence of nuclear mitochondrial sequences (NUMTs).

**Fig. 3:**
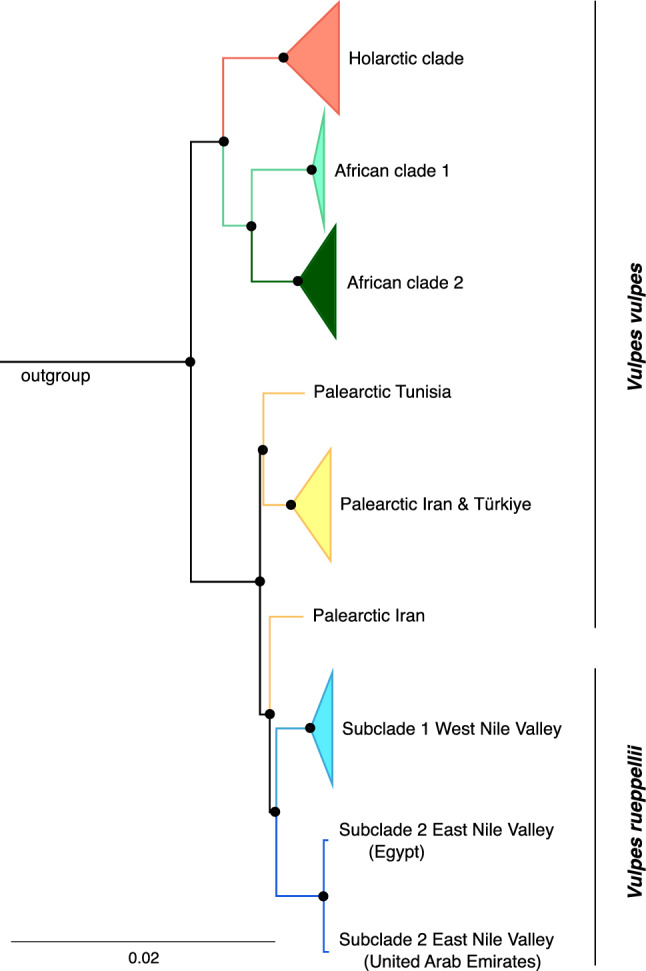
Bayesian inference tree of mitogenomes of red and Rüppell’s foxes estimated in MrBayes. Scale bar represents 2% divergence. Bayesian posterior probabilities (BPP) are indicated in the nodes with circles, where black circles represent high probabilities (BPP > 0.95).

The paraphyletic pattern previously observed in red fox was supported by both mitogenomes and partial mtDNA fragments (BPP=1.0, Figure 3 and Figure S2). A total of 8 mitogenomes recovered from red fox samples, including the five mitogenomes generated from Türkiye, grouped within Rüppell’s fox cluster (Figure 3), referred here as Palearctic. Phylogenetic analysis recovered two distinct lineages from red fox within this Palearctic cluster: one comprising 6 haplotypes among 7 mitogenomes from Tunisia, Iran, and Türkiye, and another represented by a single haplotype from Iran (Table 1 and Figure 3).

**Table 1:** Genetic diversity summary statistics based on complete mitogenomes of red and Rüppell’s foxes, including number of samples (N), number of polymorphic (segregating) sites (S), number of haplotypes (h), haplotype (Hd) and nucleotide (π) diversities with respective standard deviation (SD).

**Species**	**Lineages**	**N**	**S**	**h**	**Hd ± SD**	**π ± SD**
*Vulpes vulpes*	Holarctic clade	18	426	18	1.000 ± 0.019	0.0059 ± 0.0003
	African clade 1	8	33	6	0.893 ± 0.111	0.0008 ± 0.0001
	African clade 2	22	244	22	1.000 ± 0.014	0.0035 ± 0.0003
	Palearctic _Tunisia, Iran & Türkiye_	7	210	6	0.952 ± 0.096	0.0048 ± 0.0012
	Palearctic _Iran_	1	-	-	-	-
*Vulpes rueppellii*	Subclade 1 _West Nile Valley_	26	203	21	0.985 ± 0.014	0.0020 ± 0.0001
	Subclade 2 _East Nile Valley_	2	13	2	1.000 ± 0.500	0.0008 ± 0.0004

Based on partial mtDNA, a total of 103 haplotypes sampled from red fox clustered with Rüppell’s fox, previously referred to as “Palearctic basal haplotypes”^17^ and potentially representing introgressed haplotypes, despite reduced phylogenetic resolution due to the limited fragment size (Figure S2). Phylogenetic analysis recovered an extensive polytomy with these haplotypes and three Rüppell’s fox subclades (Figure S2). These haplotypes are widely distributed across North Africa, the Middle East, and East Asia, including the Anatolian Peninsula, Tajikistan, northeastern Pakistan, the Indian subcontinent, and Japan (Figure 2). From our extensive sampling across Türkiye, we observed that 10 % of the sampled haplotypes (5 out 49) grouped with those of Rüppell’s fox. Palearctic haplotypes were found in seven individuals distributed across Türkiye, including its eastern, southern and westernmost regions, including the European part of the country, yielding an overall prevalence of 8.75% (Figure S1).

As previously reported, both Rüppell’s and red foxes exhibit clear geographic structuring of their mitochondrial lineages, as evidenced by the phylogenetic trees (Figure 3 and Figure S2) and median-joining networks (Figure S3 and S4). In contrast, the Palearctic haplotypes show no obvious geographic patterns. Nonetheless, several small subclusters of “Palearctic basal haplotypes” were recovered with support, including subclades from: i) Japan, ii) Egypt and Tunisia, iii) Egypt, Sudan and Türkiye, iv) Iran and Iraq, and v) Türkiye (BPP > 0.95, Figure S2), suggesting their shared ancestry.

Genetic distances were largely similar between the Palearctic and red fox lineages (2.1 ± 0.1%, Table S2), and between Rüppell’s and red foxes (2.2 ± 0.1%, Table S2). In contrast, the genetic distance between the Palearctic lineage and Rüppell’s fox is much lower (0.95 ± 0.1%, Table S3), resembling that observed between the North African and Middle East subclades of Rüppell’s fox (0.9 ± 0.1%, Table S3). The Palearctic lineage from Tunisia, Iran, and Türkiye exhibited similar genetic diversity to the remaining clades of red and Rüppell’s fox (Table 1).

**Fig. 4:**
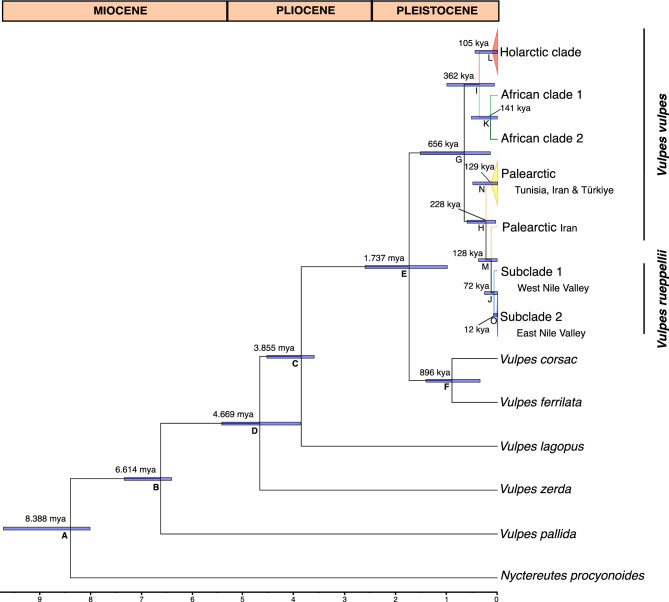
Chronogram estimated from mitogenomes using a fossil and secondary calibration approach and relaxed molecular clock implemented in BEAST. Numbers in nodes correspond to median posterior estimates of the time to the most recent common ancestor (tMRCA) given in million years ago (mya) and thousand years ago (kya) and bars correspond to 95% HPD intervals (see Table 2). Letters in nodes correspond to a list of estimated divergence times and 95% HPD (see Table 2). Letters in bold correspond to calibration points (see text). Timescale at bottom is in mya and geological timescale is shown at top.

Despite the wide confidence intervals, the estimated time to the most recent common ancestor (tMRCA) of all mitochondrial clades of red and Rüppell’s foxes falls within the Pleistocene, at approximately 656 kya (95% highest posterior density HPD = 143–1,516 kya). The red fox subsequently split into North Africa and Holarctic lineages around 362 kya (95% HPD = 64–996 kya), followed by dispersal of the Holarctic lineage into North Africa ^22^. The tMRCA between Rüppell’s fox and the Palearctic lineages was estimated at 228 kya (95% HPD = 40–601 kya). More recently, Rüppell’s fox diversified into West and East Nile Vallery lineages at approximately 72 kya (95% HPD= 5–258 kya) (Figure 4, Table 2).

**Table 2:** Posterior estimates of time to the most recent common ancestor (tMRCA) of mitogenomes of *Vulpes*, and 95% highest posterior density intervals (95% HPD) obtained in BEAST analysis. Node names are the same as in Figure 4. Time is given in thousand years ago (kya).

**Node**	**Median**	**95% HPD**
A	8,388	8,000–9,702
B	6,614	6,400–7,327
C	3,854	3,600–4,530
D	4,669	3,873–5,415
E	1,737	988–2,602
F	896	345–1,407
G	656	143–1,516
H	228	40–601
I	362	64–996
J	72	5–258
K	141	3–518
L	105	1–444
M	128	10–375
N	129	2–487
O	12	0–80

## Discussion

The ancient mitochondrial lineages carried by red fox, first described by as “Palearctic basal haplotypes”^17^ and later identified as the result of introgression by^12^ were, as expected, recovered in our study clustered within a single clade with Rüppell’s fox. Although the combined dataset of partial mitochondrial haplotypes recovered an extensive polytomy – likely due to a lack of segregating sites that hinder clear lineage sorting – the phylogenetic analysis of mitogenomes recovered a well-supported and structured topology within the previously described “Palearctic” clade^11,12^. Within this clade, we identified two independent mitochondrial lineages carried by red foxes, as well as a novel lineage of Rüppell’s fox from the UAE, which emerged as sister to the Egyptian lineage within the East Nile Valley clade.

The phylogenetic inference of Rüppell’s fox as sister to Palearctic haplotypes, the close genetic distance between Rüppell’s foxes and these haplotypes, and the estimated tMRCA between the Palearctic haplotypes and each fox species, provide support for the hypothesis of unidirectional mitochondrial introgression occurring from Rüppell’s fox into the red fox. This interpretation is further reinforced when considered alongside ecological features, biogeographic context, and previously published nuclear genomic data (e.g., candidate genes for desert adaptation introgressed into red fox^22^). While these lines of evidence do not exclude alternative scenarios, their convergence strengthens the plausibility of unidirectional introgression from Rüppell’s fox into the red fox. Future quantitative model comparison methods, such as approximate Bayesian computation (ABC), would be valuable for explicitly testing multiple plausible models with different directions of introgression.

This mitochondrial introgression may have been facilitated by shifts in ecological conditions and asymmetrical reproductive behaviour. First, past climate oscillations that translated in cycles of pluvial and interpluvial periods in many regions of the planet, including the Middle East and North Africa^26–28^ allowed the generalist red fox to expand its range into more arid regions occupied by Rüppell’s fox, favouring gene flow^22^. Under this scenario, introgression is expected to occur mostly from the local to the invading species, particularly at genetic markers experiencing reduced gene flow, such as the mitochondrial DNA^4^. The opposite direction of introgression does not appear to have occurred during interpluvial periods, possibly due to Rüppell’s fox being a strict desert specialist. Second, following the male competition hypothesis^24^, the larger red fox males, which outweighs Rüppell’s fox males, both historically and at the present^18–20^, may have had reproductive advantage in accessing Rüppell’s fox females, favouring unidirectional crosses and the transfer of Rüppell’s fox mitochondrial DNA into the red fox. Third, under the frequency-dependent assortative mating hypothesis^25^, the lower abundance of Rüppell’s fox compared to red fox in sympatric areas^19,20^ may have further facilitate the introgression of its mitochondrial genome into red fox.

The detection of two well-supported and divergent Palearctic mitochondrial lineages suggests that introgression most likely occurred at least twice. These lineages are paraphyletic, indicating that they are unlikely to have originated from a single introgressed ancestral lineage. These events likely preceded the red fox colonization of North Africa around 78 kya^22^, as one of the Palearctic lineages is shared between Eurasian and North African red fox populations (Türkiye, Iran and Tunisia), while the other appears to be restricted to Eurasia (Iran). Importantly, the assignment of Palearctic haplotypes identified solely from partial mitochondrial sequences remains uncertain, and may correspond to the widely distributed lineage or potentially reflect additional, yet unconfirmed, introgression events that warrant further investigation.

Although both mitochondrial Palearctic lineages cluster with Rüppell’s fox, neither is nested within any of the extant Rüppell’s fox lineages, further suggesting that Palearctic haplotypes originated from now-extinct (ghost) mitochondrial lineages of Rüppell’s fox. These findings are consistent with cases of ghost introgression or introgression from as-yet unsampled lineages, a phenomenon increasingly recognized in mammalian evolution^10,23,29–31^, and are further supported by divergence time estimates observed in foxes.

While supporting an older split between the two species around 656 kya (95% HPD = 143–1,516 kya), consistent with both mitochondrial tMRCA and whole-genome divergence time estimates from a previous study^22^, our estimates suggest that the coalescent time of Rüppell’s fox and Palearctic lineages is approximately 228 kya (95% HPD = 40–601 kya), preceding the coalescent time of all Rüppell’s fox mitochondrial lineages, estimated at 72 kya (95% HPD=5–258 kya). This pattern further supports the hypothesis of ancient introgression from now-extinct (ghost) mitochondrial lineages of Rüppell’s fox.

Notably, the mitochondrial Palearctic haplotypes (previously referred to as “Palearctic basal haplotypes”^17^) are not restricted to a specific part within the red fox range. Despite apparently predominant at the southern fringe of the species distribution, where the ranges of the two species overlap^15,16^, they are also present across a broader geographic range, including the Anatolian Peninsula, Tajikistan, Northeast Pakistan, the Indian subcontinent, and Japan. With the exception for Japan, these regions geographically surround the contact zone between the two species, suggesting that once introgressed into the red fox mitochondrial gene pool, these Palearctic haplotypes could have subsequently spread through demographic expansion and gene flow, eventually reaching regions far from the original zone of contact.

Importantly, Palearctic haplotypes occur at low frequency, as highlighted by our comprehensive sampling in the Anatolian Peninsula, suggesting that their detection requires extensive and geographically structured surveys of local genetic diversity. Their rarity is consistent with introgression remaining limited in the face of substantial intraspecific gene flow within populations^5^. Nonetheless, the occurrence of these haplotypes across geographically distant regions suggests that similar low-frequency haplotypes may also be present in other areas that have not yet been thoroughly sampled.

This case of ancient mitochondrial introgression between two sister species of the genus *Vulpes* represents a compelling empirical example of how multiple, complementary genetic and ecological hypotheses can be integrated to reconstruct the evolutionary and demographic patterns of ghost maternally inherited molecular lineages. Moreover, it serves as another illustration of the evolutionary complexity within canids, a group that continues to offer fascinating insights into hybridization, adaptation, and lineage diversification.

## Methods

### Data generation and compilation

We built two datasets both using newly generated and publicly available sequences: i) one dataset with seven mitogenomes of red foxes from Türkiye and one mitogenome of Rüppell’s fox from the UAE newly generated (see below) together with 53 mitogenomes of red foxes and 27 Rüppell’s fox retrieved from literature^11,22^ (see Figure 1 and Table S1); and ii) another dataset with 222 mtDNA cytochrome b and D-loop partial sequences of red foxes from Europe, Iran and North Africa, including 80 newly generated sequences from Türkiye, 57 sequences of Rüppell’s foxes from North Africa and the UAE^12,22^, and 41 sequences identified as “Palearctic basal haplotypes” of red fox^17^ (Table S2).

The pale fox *Vulpes pallida* (Cretzschmar, 1826), fennec fox *Vulpes zerda* (Zimmermann, 1780), Tibetan fox *Vulpes ferrilata* Hodgson, 1842, corsac Fox *Vulpes corsac* (Linnaeus, 1768) and arctic fox *Vulpes lagopus* Linnaeus, 1758 were used as outgroups in all analysis.

### DNA extraction and sequencing of mitogenomes

Genomic DNA was extracted using the Qiagen DNeasy Blood & Tissue Kit, following the manufacturer’s protocol, and eluted in EB buffer (Qiagen). Genomic DNA samples were sheared to an average fragment size of 350 bp using the Bioruptor sonicator (Diagenode), followed by double size selection using AMpure XP beads (Beckman Coulter), following the Illumina’s Truseq DNA PCR-Free protocol.

Sequencing libraries were built using KAPA Hyper Prep Kit (KAPA Biosystems), following manufacturer’s protocol, with one quarter reaction volumes. Dual-indexing PCR was performed with TruSeq UD indexes (Illumina) using KAPA HiFi Hotstart Ready Mix (KAPA Biosystems). Libraries were pooled in equimolar ratios and paired-end sequenced (2 x 150 bp) on a single S4 lane of the Illumina NovaSeq6000 Sequencing System. Samples were sequenced to an expected average raw coverage of 5x of whole genome sequencing, accounting for 20% of loss due to sequencing duplicates.

Raw read quality was assessed using FastQC^32^. Adapter sequences, short reads (<30 bp), and low-quality bases (Q<20) were removed with AdapterRemoval v2^33^, and overlapping reads were collapsed with the same software. The retained read pairs were aligned to a reference genome consisting of the complete red fox nuclear genome (VulVul3, GCF_048418805.1) concatenated with complete mitochondrial genome (OZ067330.1)^34^ using Burrows-Wheeler Aligner (BWA 0.7.17-r1198-dirty) with the mem algorithm^35^. Mapped reads were extracted and processed in SAMtools 1.5^36^, excluding those with mapping qualities below 20. PCR duplicates were removed using Picard v2.23 (http://broadinstitute.github.io/picard/). To minimize the inclusion of nuclear mitochondrial sequences (NUMTs), only reads that mapped exclusively to the mitochondrial genome were retained with the SAMtools 1.5^36^. Consensus mitochondrial sequences were reconstructed for each sample using ANGSD^37^, considering only positions with a minimum sequencing depth of 10x. Furthermore, because NUMTs may pose challenges for phylogenetic inference, we carefully inspected all reconstructed sequences, specifically screening for premature stop codons^38^.

Three red fox mitogenomes were excluded due to alignment ambiguities, resulting in a final dataset of 85 mitogenomes used in downstream analysis. The newly generated mitogenomes were aligned to previously published red fox mitogenomes^22^, excluding the hypervariable region (D-loop) due to alignment ambiguities using Geneious Prime® 2023.2.1.

### DNA extraction, amplification and sequencing of mitochondrial DNA fragments

Genomic DNA was extracted from 80 red fox tissue samples, collected from roadkills in 70 localities across Türkiye (Table S2 and Figure S1), using the DNeasy Tissue Kits (Qiagen).

A mitochondrial DNA fragment, including the cytochrome b gene (cyt-*b*, 1140 bp) and the 5′ end (the hypervariable region I) of the control region (304 bp, excluding indels), was amplified by Polymerase Chain Reaction (PCR) with specific primers (see Table S3 for primers details). PCR products were sequenced in both directions using the Big-Dye Terminator v. 3.1 Cycle Sequencing protocol (Applied Biosystems). Electropherograms were checked and aligned, and ambiguous bases were resolved manually using Sequencher v. 4.5 (Gene Codes Corp.). These sequences were then aligned with additional publicly available data and trimmed to a consensus fragment size of 637 bp (332 bp of cytb and 305 of control region) to minimize missing data, using Geneious Prime® 2023.2.1 (https://www.geneious.com).

### Phylogenetic inference and genetic differentiation

Bayesian inference (BI) was performed in MrBayes v3.2.7^39^ using the two datasets separately, with the best-fit substitution models selected according to the Bayesian information criterion in jModelTest2^40^: the GTR+I+G model for mitogenomes and the HKY+I+G model for partial mitochondrial sequences. Two independent analyses of Metropolis-coupled Markov chain Monte Carlo (MCMC) were run simultaneously for 10x10^6^ generations. Trees were sampled every 500 generations until MCMC became stationary, i.e., when the standard deviation of split frequencies was below 0.01, indicating good convergence. A 50% majority rule consensus tree was obtained after discarding of 25% of the sampled trees as burn-in to generate Bayesian posterior probabilities (BPPs). Consensus trees were visualized in FigTree v1.4.4^41^.

Genetic diversity parameters, including DNA polymorphism, number of haplotypes, haplotype and nucleotide diversities were estimated for the mitogenome dataset using DnaSP v. 6^42^. A median-joining network was constructed for both the mitogenome and partial mitochondrial sequence datasets using PopART^43^. Genetic distances were estimated using Kimura 2-parameter (K2P,^44^) in MEGA 12^45^.

### Time to the most recent common ancestor estimation

The time to the most recent common ancestor (tMRCA) was estimated using mitogenomes, and a calibrated approach implemented in BEAST 1.10.4^46^, assuming a Yule speciation model^47^ and an uncorrelated relaxed molecular clock, which is suitable for estimating interspecific relationships^48^. MCMC run was set up in BEAUti using the substitution model GTR+I+G, as selected by jModelTest2^40^ (see above) and uncorrelated relaxed molecular clock with lognormal distribution.

We used both fossil and secondary calibrations, following the approach of Rocha et al.^22^. Fossil calibration points were set using a gamma prior as follows: A) the split between the tribes Canini, represented by the common racoon dog (*Nyctereutes procyonoides*), and Vulpini was set with an offset of 8, corresponding to the earliest record of Eocyon^49^, shape=1 and scale=1.4 (95% HPD 8.07–12.19 mya), based on the approximate split between the tribes Canini and Vulpini^8^; B) the split between the pale fox *V. pallida* and the remaining fox species was set with an offset of 6.4, shape=1 and scale=0.4 (95% HPD 6.42–7.6 mya) corresponding to the radio-metrical dating of the Toros-Menalla fauna (7.6 to 6.4 mya) which includes the oldest fossil of the Old world *Vulpes riffautae*^50,51^; and C) the split between the arctic fox *V. lagopus* and the remaining fox species was set with an offset of 3.6, shape=1 and scale=0.6 (95% HPD 3.63–5.10 mya), corresponding to the earliest arctic fox ancestor, *Vulpes qiuzhudingi*, from the Zanda Basindated, dated between 5.0 and 3.6 mya^50^. Secondary calibration points were set using a normal prior as follows: D) the tMRCA between the fennec fox and other fox species was set to 4.72 ± 0.5 mya^52^; E) the tMRCA between the corsac/Tibetan fox (*V. corsac/V. ferrilata*) and the remaining *Vulpes* species was set to 1.8 ± 0.5 mya; and F) the tMRCA between the corsac and Tibetan fox was set to 1.02 ± 0.3 mya^52^. The remaining tree nodes were set according to a previous phylogeny^22^ and our BI results (see above).

We performed two independent MCMC runs of 500 × 10^6^ generations each, sampled every 50 × 10^3^ generations. Convergence of the MCMC chains was verified in Tracer v.1.7^53^, ensuring that all effective sample size (ESS) values were higher than 200. The resulting trees were combined using LogCombiner, and the consensus tree was generated in TreeAnotator after a burn-in of 25%. Consensus trees were visualized in FigTree 1.4.4^41^.

## Supplementary Information

Supplementary Information 1.

Supplementary Information 2.

## Data Availability

The datasets generated during the this study are available in the GenBank repository, under the accession numbers: PV802399–PV802404 and PV800565–PV800662.
