# Supplementary material for: Multiple introgression events from ghost Rüppell’s fox mitochondrial lineages into red fox
Source: Sci Rep. 2026 Mar 28;16:10772. doi: 10.1038/s41598-026-45528-8 (PMC13040000; doi:10.1038/s41598-026-45528-8)
Supplement: Supplementary file 1 — Supplementary Information 1. [file 41598_2026_45528_MOESM1_ESM.docx]

**Supplementary material**

**Multiple introgression events from ghost Rüppell’s fox mitochondrial lineages into red fox**

Rita Gomes Rocha^1,2,*^, Ali Adan Hassan^3^, Sadık Demirtaş^4^, Mariana Meneses-Ribeiro^1,2,5^, Joana L. Rocha^6^, İslam Gündüz^3,#^, Raquel Godinho^1,2,5,7,**,#^

^1^InBIO Laboratório Associado, CIBIO–Centro de Investigação em Biodiversidade e Recursos Genéticos, Universidade do Porto, Vairão, Portugal

^2^BIOPOLIS Program in Genomics, Biodiversity and Land Planning, Centro de Investigação em Biodiversidade e Recursos Genéticos (CIBIO), Vairão, Portugal

^3^Department of Biology, Faculty of Sciences, Ondokuz Mayis University, Samsun, Türkiye

^4^Department of Molecular Biology and Genetics, Faculty of Sciences, Ondokuz Mayıs University, Samsun, Türkiye

^5^Departamento de Biologia, Faculdade de Ciências, Universidade do Porto, Porto, Portugal

^6^Department of Integrative Biology, University of California Berkeley, Berkeley, CA, USA

^7^Department of Zoology, University of Johannesburg, Johannesburg, South Africa

#co-senior authors

**Correspondence**:

*Rita Gomes Rocha

[rgrocha@cibio.up.pt](mailto:rgrocha@cibio.up.pt)

**Raquel Godinho

[rgodinho@cibio.up.pt](mailto:rgodinho@cibio.up.pt)

**Table S1.** Data of all mitogenomes used in this study, including sample ID, species identification, mitochondrial DNA lineage, location (country and coordinates) and source (see supplementary excel file). Data retrieved from [1,2].

**Table S2.** Data of all partial mitochondrial DNA fragments used in this study, including sample ID, species identification, mitochondrial DNA lineage, location (country and coordinates) and source (see supplementary excel file). Data retrieved from [1,3,4].

**Table S3.** Information on primers used in this study to amplify (*V. vulpes*: L14163_Vv and H15765; *V. rueppellii*: L14163_Vr and H15765) and sequencing (*V. vulpes*: L14163_Vv, L15035_IG, H15187_IG and H15762_IG; *V. rueppellii*: L14163_Vr, L15035_IG, H15187_IG and H15762_IG) a fragment of mitochondrial DNA of foxes.

| **Primer** | **Sequence (5’ – 3’)** | **Source** |
| --- | --- | --- |
| L14163_Vv | GATATGAAAAATCACCGTTG | Modified from L14727-SP [5] |
| L14163_Vr | TGACATGAAAAATCATCGTTG | Modified from L14727-SP [5] |
| H15765 | CCTGAGGTAAGAACCAGATG | Modified from H16498 [6] |
| L15035_IG | CCTATGCYATTCTCCGGTCCA | This study |
| H15187_IG | GGCTGCCCTCCGATTCAAGTTAAG | This study |
| H15762_IG | CCTGAGGTAAGAACCAGATGCCA | This study |

**Table S4.** Estimates of pairwise genetic distances between clades of *Vulpes* as determined using Bayesian inference (Fig. 1). Analyses were conducted using 96 mitogenomes in a final data set comprising 16,116 positions, and using the Kimura 2-parameter model [7], implemented in MEGA 12 [8]. The number of base substitutions per site from averaging over all sequence pairs between groups are shown. Standard error estimate(s) are shown above the diagonal.

|  |  | *V. vulpes* | *V. rueppellii* | | *V. vulpes* | | | *V. ferrilata* | *V. corsac* | *V. lagopus* | *V. zerda* | *V. pallida* |
| --- | --- | --- | --- | --- | --- | --- | --- | --- | --- | --- | --- | --- |
|  |  | Palearctic | Subclade 1 North Africa | Subclade 2 Middle East | Holarctic clade | African clade 1 | African clade 2 |  |  |  |  |  |
| *V. vulpes* | Palearctic |  | 0.001 | 0.001 | 0.001 | 0.001 | 0.001 | 0.002 | 0.002 | 0.003 | 0.003 | 0.003 |
| *V. rueppellii* | Subclade 1 North Africa | 0.010 |  | 0.001 | 0.001 | 0.001 | 0.001 | 0.002 | 0.002 | 0.003 | 0.003 | 0.003 |
|  | Subclade 2 Middle East | 0.009 | 0.009 |  | 0.001 | 0.001 | 0.001 | 0.002 | 0.002 | 0.002 | 0.003 | 0.003 |
| *V. vulpes* | Holarctic clade | 0.021 | 0.022 | 0.023 |  | 0.001 | 0.001 | 0.002 | 0.002 | 0.003 | 0.003 | 0.003 |
|  | African clade 1 | 0.021 | 0.022 | 0.022 | 0.017 |  | 0.001 | 0.002 | 0.002 | 0.003 | 0.003 | 0.003 |
|  | African clade 2 | 0.021 | 0.022 | 0.022 | 0.017 | 0.012 |  | 0.002 | 0.002 | 0.003 | 0.003 | 0.003 |
| *V. ferrilata* |  | 0.076 | 0.076 | 0.077 | 0.076 | 0.075 | 0.076 |  | 0.002 | 0.003 | 0.003 | 0.003 |
| *V. corsac* |  | 0.076 | 0.076 | 0.077 | 0.076 | 0.074 | 0.075 | 0.036 |  | 0.003 | 0.003 | 0.003 |
| *V. lagopus* |  | 0.094 | 0.093 | 0.094 | 0.094 | 0.094 | 0.093 | 0.098 | 0.099 |  | 0.003 | 0.003 |
| *V. zerda* |  | 0.112 | 0.112 | 0.111 | 0.111 | 0.111 | 0.112 | 0.113 | 0.112 | 0.115 |  | 0.003 |
| *V. pallida* |  | 0.112 | 0.113 | 0.112 | 0.112 | 0.113 | 0.113 | 0.114 | 0.114 | 0.117 | 0.103 |  |

**Figure S1.** Geographic location of all red foxes sampled across Türkiye. Colours represent maternal lineages obtained in the phylogenetic analysis (see Fig. S4). Dashed and grey areas represent the distribution of red and Rüppell’s foxes, respectively, obtained from IUCN [9,10].

**Figure S2.** Bayesian inference tree of partial mitochondrial DNA haplotypes (cyt-*b* and control region) of red and Rüppell’s foxes estimated in MrBayes using the combined dataset of 289 shorter fragments (637 bp). Scale bar represents 2% divergence. Bayesian posterior probabilities (BPP) are indicated in the nodes with circles, where black circles represent high probabilities (BPP > 0.95) and white circles represent low probabilities (BPP < 0.95).

**Figure S3.** Median-joining network of 87 mitogenomes of red and Rüppell’s foxes. Circle size is proportional to the frequency of each haplotype. Dashes and numbers in the branches correspond to nucleotide substitutions.

**Figure S4.** Median-joining network of 289 partial mitochondrial DNA sequences (cyt-*b* and control region) representing 103 haplotypes of red and Rüppell’s foxes. Circle size is proportional to the frequency of each haplotype. Dashes in the branches correspond to nucleotide substitutions.

**References**

1. Rocha, J. L. *et al.* North-African fox genomes show signatures of repeated introgression and adaptation to life in deserts. *Nat Ecol Evol* (2023).

2. Basuony, A. E., Saleh, M. & Hailer, F. Mitogenomic analysis of Rüppell’s fox (Vulpes rueppellii) confirms phylogenetic placement within the Palaearctic clade shared with its sister species, the red fox (Vulpes vulpes). *Mitochondrial DNA A DNA Mapp Seq Anal* **0,** 1–7 (2024).

3. Basuony, A. E. *et al.* Paraphyly of the widespread generalist red fox ( Vulpes vulpes ): introgression rather than recent divergence of the arid-adapted Rüppell ’ s fox ( Vulpes rueppellii )? *Biological Journal of the Linnean Society* **138,** 453–469 (2023).

4. Statham, M. J. *et al.* Range-wide multilocus phylogeography of the red fox reveals ancient continental divergence, minimal genomic exchange and distinct demographic histories. *Mol Ecol* **23,** 4813–4830 (2014).

5. Jaarola, M. & Searle, J. B. Phylogeography of field voles (Microtus agrestis) in Eurasia inferred from mitochondrial DNA sequences. *Mol Ecol* **11,** 2613–2621 (2002).

6. Meyer, A., Kocher, T. D., Basasibwaki, P. & Wilson, A. C. Monophyletic origin of Lake Victoria cichlid fishes suggested by mitochondrial DNA sequences. *Nature* **347,** 550–553 (1990).

7. Kimura, M. A simple method for estimating evolutionary rates of base substitutions through comparative studies of nucleotide sequences. *J Mol Evol* **16,** 111–120 (1980).

8. Kumar, S. *et al.* MEGA12: Molecular Evolutionary Genetic Analysis Version 12 for Adaptive and Green Computing. *Mol Biol Evol* **41,** msae263 (2024).

9. Hoffmann, M. & Sillero-Zubiri, C. Vulpes vulpes (amended version of 2016 assessment). *The IUCN Red List of Threatened Species 2021: e.T23062A193903628.* (2021).

10. Mallon, D., Murdoch, J. D. & Wacher, T. Vulpes rueppelli. *The IUCN Red List of Threatened Species 2015: e.T23053A46197483* (2015).
